# Supplementary material for: Content-rich biological network constructed by mining PubMed abstracts
Source: BMC Bioinformatics. 2004 Oct 8;5:147. doi: 10.1186/1471-2105-5-147 (PMC528731; doi:10.1186/1471-2105-5-147)
Supplement: Additional File 2 — The original results of the above study (non-essential files are deleted to keep the file size under the limit set by BMC bioinformatics). [file 1471-2105-5-147-S2.bz2 › chilibotAdditionalFile2/dip05/681ID11493686E17996/html/DNAB_TUS.html]

 


 **DNAB** and **TUS** 
  
Found 11 abstracts in PubMed, retrieved 05.  
 

 What does Google say? 
 PDF only 
| .edu only 

---

**Interactive relationship** (e.g. stimulation, inhibition, etc)

**Neutral relationship**- We show further that replication termination involves protein protein interaction between  **Tus**  and  **DnaB**  at a critical region of  **Tus**  protein, called the L1 loop.  Ref: 11493686 Proc Natl Acad Sci U S A, 2001
- Several mutations located in the L1 loop region not only reduced the protein protein interaction but also eliminated or reduced the ability of the mutant forms of  **Tus**  to arrest  **DnaB**  at a Ter  [ **TUS** ]  site.  Ref: 11493686 Proc Natl Acad Sci U S A, 2001
- Using yeast forward and reverse two hybrid analyses, we have discovered that the replication terminator protein  **Tus**  of Escherichia coli physically interacts with  **DnaB**  helicase in vivo.  Ref: 11493686 Proc Natl Acad Sci U S A, 2001
- They appeared to bind Ter  [ **TUS** ]  DNA normally but were ineffective in fork arrest as ascertained by in vitro Escherichia coli  **DnaB**  helicase and replication assays.  Ref: 10064700 J Mol Biol, 1999

**Non-interactive relationship** (e.g. studied together, co-existance, homology, etc.)

- At least one mutation, E49K, significantly reduced  **Tus**   **DnaB**  interaction and almost completely eliminated the contrahelicase activity of  **Tus**  protein in vitro without significantly reducing the affinity of the mutant form of  **Tus**  for Ter  [ **TUS** ]  DNA, in comparison with the wild type protein.  Ref: 11493686 Proc Natl Acad Sci U S A, 2001
